# Supplementary material for: A Global Screen for Assembly State Changes of the Mitotic Proteome by SEC-SWATH-MS
Source: Cell Syst. 2020 Feb 26;10(2):133–155.e6. doi: 10.1016/j.cels.2020.01.001 (PMC7042714; doi:10.1016/j.cels.2020.01.001)

P17028 | ZNF24_HUMAN | ZNF24 KOX17 ZNF191 ZSCAN3
Monomer MW [kDa]: 42.155 Monomer expected elution fraction: 48

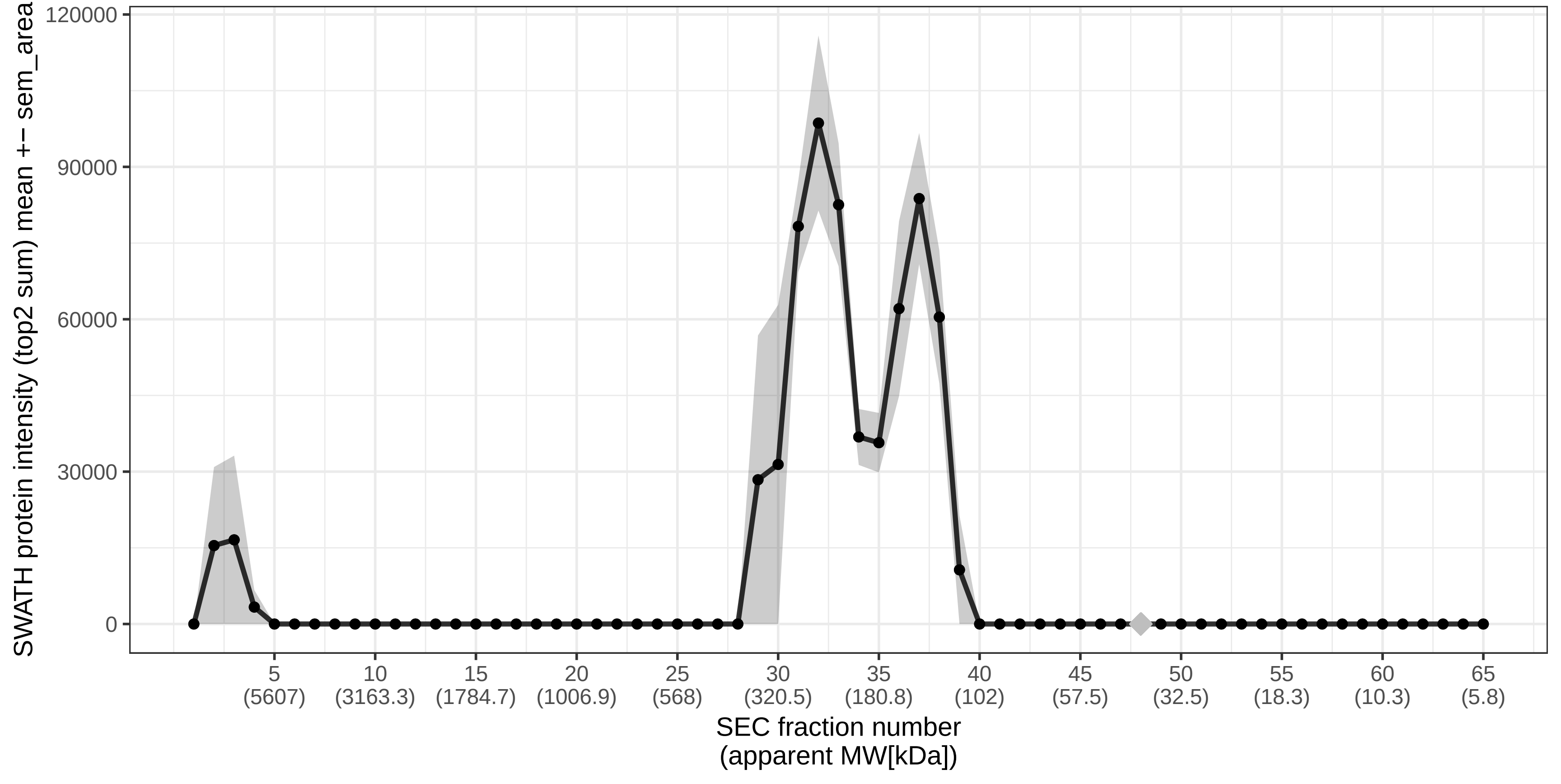

Supplement: Data S1. SEC-SWATH-MS Protein Chromatograms, Related to Figure 1 [file mmc6.zip › SECchrom_P17028_ZNF24_HUMAN_ZNF24_KOX17_ZNF191_ZSCAN3.pdf]
